# High-functioning depression: a hidden burden demanding clinical recognition

**DOI:** 10.1192/bjb.2025.10193

**Published:** 2026-04

**Authors:** Promise U. Okereke, Chukwuemeka V. Umeh, Wisdom O. Okereke, Egide Ndayambaje, Christian C. Obetta, Onyedikachi F. Uzor, Olanrewaju J. Oduola

**Affiliations:** 1 Faculty of Dentistry, College of Medicine, University of Nigeria, Enugu, Nigeria; 2 Global Leadership Dentistry and Sciences, Washington, District of Columbia, USA; 3 Alliance for Oral Health Across Borders, New York, New York, USA; 4 Faculty of Clinical Sciences, College of Medicine, University of Nigeria, Enugu, Nigeria; 5 Sheffield Teaching Hospitals NHS Foundation Trust, Sheffield, UK; 6 Ideal Dental Foundation International, Abuja, Nigeria; 7 College of Medicine and Health Sciences, University of Rwanda, Kigali, Rwanda; 8 Research Department, OLGNova, Kigali, Rwanda; 9 Department of Public Health, Oxford Brookes University, Oxford, UK; 10 Department of Periodontology and Community Dentistry, University College Hospital, Ibadan, Nigeria

**Keywords:** Depressive disorders, diagnosis and classification, mental health services, psychotherapy, clinical outcome measures

## Abstract

High-functioning depression (HFD) describes individuals experiencing persistent depressive symptoms, such as low mood and emotional exhaustion, while maintaining outward success. Owing to preserved functionality, the underlying distress is often unnoticed, misattributed or suppressed. HFD challenges existing psychiatric frameworks, delays diagnosis and increases the risk of progression to major depressive disorder and suicidality. Current screening tools may lack sensitivity, and stigma can lead to disengagement from therapy. Expanded diagnostic awareness, improved clinician training and culturally attuned care are essential for recognising and validating internal suffering in this overlooked population.

High-functioning depression (HFD) is an increasingly recognised presentation in psychiatric practice, although it remains diagnostically undefined.^1^ The term ‘high-functioning depression’ has appeared intermittently in clinical discourse and popular psychology since the late 20th century, often as an informal descriptor for individuals who meet criteria for persistent depressive disorder while maintaining social and occupational roles.^2^ Although not formally recognised in DSM-5 or ICD-11, just like some other atypical depressive conditions, HFD is becoming increasingly acknowledged in clinical commentary and practice discussions.^1,2^ Individuals affected often experience enduring symptoms of depression while continuing to perform professionally and socially, thus maintaining outward success and social functionality.^2^ These individuals may appear well-adjusted, productive and emotionally stable, yet silently endure persistent low mood, fatigue, irritability and emotional detachment.^1,2^ Despite substantial internal distress, their ability to meet certain obligations, such as professional, academic or familial obligations, often obscures the severity of their symptoms and therefore their internal psychological burden is often unnoticed, unacknowledged, suppressed or dismissed.^3^ As a result, HFD remains a blind spot in psychiatric diagnostics, underpinned by a culture that frequently equates visible functionality with mental wellness.

Although HDF is not formally recognised in DSM-5 and ICD-11, it closely overlaps with persistent depressive disorder (dysthymia), albeit with fewer observable functional impairments. In clinical contexts, it challenges the assumption that depression necessarily manifests as noticeable dysfunction.^3,4^ For mental health services globally, this creates a diagnostic lacuna with implications for care, public health and policy.

## A challenge to diagnostic assumptions

Current psychiatric frameworks tend to associate depression with functional impairment.^3,4^ In contrast, individuals with HFD often retain high performance in professional or caregiving roles while concealing symptoms. Many may describe themselves as ‘just tired’ or ‘coping under pressure’. In such contexts, clinicians may misattribute signs of distress to occupational stress, personality traits such as perfectionism, or situational burnout.^5,6^

Furthermore, conventional screening tools such as the Patient Health Questionnaire-9 or General Anxiety Disorder-7 may inadequately capture subthreshold but persistent symptoms in individuals who minimise their distress.^7^ This diagnostic ambiguity may delay intervention until the individual reaches a point of collapse, often presenting in crisis or through somatic complaints.

The challenge is compounded in high-functioning populations such as students, physicians, carers and executives, who are socially and professionally incentivised to appear emotionally self-sufficient.^2^

## The paradox of success and silence

The sociocultural perception of depression is often tied to dysfunction.^8^ However, high-functioning individuals may embody the very traits society rewards: resilience, ambition and self-control. For many, these traits mask vulnerability.^5,6^

Women, ethnic minorities and professionals in high-pressure environments may feel an implicit obligation to maintain a composed façade.^5,6,9^ Studies have found that such individuals often internalise the belief that they are ‘not unwell enough’ to seek help, particularly if their suffering is not externally visible.^8,9^

This paradox is exacerbated by mass media representations that either dramatise breakdowns or celebrate ‘toughness’ in adversity. The deaths of public figures who appeared successful on the surface highlight the limits of our current diagnostic paradigms.^9^ Yet, clinical reforms rarely follow these public reckonings. Also, the interplay of chronic stress should not be underestimated, as these stressors, be they environmental, neuroendocrinological and/or genetic, further compound the silent progression to a major depressive condition.^10,11^ It is noteworthy that HFD may be preceded and/or triggered by burnout, as their symptoms, masking and treatments most likely overlap.^12^

## Clinical and public health implications

Delayed recognition of HFD carries significant risks. Prolonged low-grade depression can evolve into major depressive episodes, increase suicidality and result in comorbid anxiety or substance use disorders.^13^ Beyond mental illness, persistent depressive symptoms are linked to cardiovascular morbidity, sleep disturbance and diminished immune function.^14^

Clinically, people with HFD may be reluctant to engage in treatment.^6^ This is not due to pharmacological resistance, but behavioural ambivalence. Patients may question the legitimacy of their need for help, feel guilt for seeking care or withdraw from therapy once external performance improves.^5,6^ Psychotherapeutic approaches such as cognitive–behavioural therapy and compassion-focused therapy may need to be adapted to engage these individuals’ high levels of autonomy, self-criticism and over-control.^15^

At a systems level, HFD contributes an invisible burden in primary care workplace environments. Mental health promotion strategies in occupational and educational settings often miss those who are technically ‘functioning’ but quietly deteriorating.^5^

## What psychiatry must address

To better serve individuals with HFD, psychiatry must evolve on four fronts.

- Broadening of screening practices: screening tools should be adapted to include emotional numbing, perfectionistic coping and internalised distress in high-performing populations.
- Recognising subthreshold depression: diagnostic frameworks such as the DSM and ICD should better acknowledge persistent, functionally masked depression and associated distress, as should practice guidelines such as those published by National Institute for Health and Care Excellence (NICE), American Psychiatric Association and World Health Organization.
- Ensuring cultural and identity awareness: training should equip clinicians to recognise how gender, culture and socio-professional identity influence help-seeking behaviours and symptom expression.
- Developing future research: further research is urgently needed to better understand this form of depression, particularly its prevalence, specific symptoms, screening/diagnostic tools and management modalities, through longitudinal studies on depression trajectories, qualitative exploration of patients’ personal experiences, and targeted interventions within high-functioning occupational groups.

## Conclusion

High-functioning depression underscores the limitations of psychiatric frameworks that equate mental illness primarily with visible dysfunction. By concealing their suffering behind competence, individuals with HFD remain largely invisible to clinicians, employers and even family members, despite enduring persistent distress. This invisibility not only delays recognition and treatment, but also reinforces the cultural myth that outward success is incompatible with mental illness.

Recognition of HFD demands a multidimensional response. Psychiatrists must be attentive to presentations where patients meet external expectations yet express chronic internal struggles. Therefore, classification systems and clinical guidelines such as DSM, ICD and NICE must evolve to account for subthreshold and masked forms of depression. At the same time, broader cultural change is needed to challenge the stigma that equates vulnerability with weakness and well-being with productivity.

Ultimately, addressing HFD requires collaboration across psychiatry, primary care, workplaces and communities. By recognising internal suffering, even when hidden behind achievement, we can close a critical gap in mental healthcare, prevent progression to more severe illness and foster a more compassionate approach to human well-being.

## Data Availability

Data availability is not applicable to this article as no new data were created or analysed in this study.

## About the authors

**Promise U. Okereke**, BDS, is a graduate of the Faculty of Dentistry, College of Medicine, University of Nigeria, Enugu, Nigeria; the Nigerian Ambassador of the Global Leadership Dentistry and Sciences, Washington, District of Columbia, USA; a student mentor with the Alliance for Oral Health Across Borders, New York, New York, USA; the Research Director, Youth Health Action Network (YOHAN) Africa, YOHAN Research Institute, Enugu, Nigeria; and the Research Lead, Ideal Dental Foundation International, Abuja, Nigeria. **Chukwuemeka V. Umeh**, MBBS, is a graduate of the Faculty of Clinical Sciences, College of Medicine, University of Nigeria, Enugu, Nigeria; and a resident doctor at the Sheffield Teaching Hospitals NHS Foundation Trust, Sheffield, UK. **Wisdom O. Okereke**, BDS, is graduate of the Faculty of Dentistry, College of Medicine, University of Nigeria, Enugu, Nigeria; the Programs and Community Engagement Officer at Ideal Dental Foundation International, Abuja, Nigeria; and President of the American Academy of Developmental Medicine and Dentistry’s Student Representative Council, Nigeria Chapter. **Egide Ndayambaje**, MBBS, is a medical student in the College of Medicine and Health Sciences, University of Rwanda, Kigali, Rwanda; and a researcher at the Research Department, OLGNova, Kigali, Rwanda. **Christian C. Obetta**, MBBS, is graduate of the Faculty of Clinical Sciences, College of Medicine, University of Nigeria, Enugu, Nigeria; and is pursuing a Master’s degree in public health in the Department of Public Health, Oxford Brookes University, Oxford, UK. **Onyedikachi F. Uzor**, MBBS, is a resident doctor with Sheffield Teaching Hospitals NHS Foundation Trust, Sheffield, UK. **Olanrewaju J. Oduola**, BDS, is a senior registrar in the Department of Periodontology and Community Dentistry, University College Hospital (UCH), Ibadan, Nigeria.
